# A randomised controlled trial to measure the effect of chest pain unit care upon anxiety, depression, and health-related quality of life [ISRCTN85078221]

**DOI:** 10.1186/1477-7525-2-39

**Published:** 2004-07-29

**Authors:** Steve Goodacre, Jon Nicholl

**Affiliations:** 1 Medical Care Research Unit, University of Sheffield, Regent Court, 30 Regent Street, Sheffield, S1 4DA, UK

## Abstract

**Background:**

The chest pain unit (CPU) has been developed to provide a rapid and accurate diagnostic assessment for patients attending hospital with acute, undifferentiated chest pain. We aimed to measure the effect of CPU assessment upon psychological symptoms and health-related quality of life.

**Methods:**

We undertook a single-centre, cluster-randomised controlled trial. Days (N = 442) were randomised in equal numbers to CPU or routine care. Patients with acute chest pain, undiagnosed by clinical assessment, ECG and chest radiograph, were recruited and followed up with self-completed questionnaires (SF-36 and HADS) at two days and one month after hospital attendance.

**Results:**

Patients receiving CPU assessment had significantly higher scores on the physical functioning (difference 5.1 points; 95% CI 1.1 to 9.0), vitality (4.6; 1.3 to 8.0), and general health (5.7; 2.3 to 9.2) dimensions of the SF-36 at two days, and significantly higher scores on all except the emotional role dimension at one month. They also had significantly lower depression scores on the HADS depression scale at two days (0.93; 0.34 to 1.51) and one month (1.0; 0.36 to 1.66). However, initially lower anxiety scores at two days (0.89; 0.21 to 1.56) were not maintained at one month (0.48; -0.26 to 1.23). CPU assessment was associated with reduced prevalence (OR 0.71; 95% CI 0.52 to 0.97) and severity (6.5 mm on 100 m visual analogue scale; 95% CI 2.2 to 10.8) of chest pain at one month, but no significant difference in the proportion of patients taking time off work (OR 0.82; 95% CI 0.54 to 1.04).

**Conclusion:**

CPU assessment is associated with improvements in nearly all dimensions of quality of life and with reduced symptoms of depression.

## Background

Acute chest pain is a common reason for emergency hospital attendance and admission. Patients with chest pain that remains undiagnosed after clinical assessment, ECG and chest radiograph pose a particular problem. They carry a low, but important risk of an acute coronary syndrome [1]. The potentially life-threatening nature of this diagnosis means that a cautious approach is often taken, with many patients being admitted to hospital for observation and investigation [2]. Yet most patients with undifferentiated chest pain do not have a coronary syndrome, whereas anxiety and psychological morbidity are common [3-5] and appear to be associated with impaired quality of life [6]. It is possible that anxiety could be influenced by the investigation and management of chest pain. If this is so, then decision analysis modelling suggests that the potential health gains that could be achieved by reducing anxiety and improving quality of life among the majority of patients who do not have an acute coronary syndrome substantially outweigh the potential health gains from detecting and treating acute coronary syndromes [7].

The chest pain unit (CPU) was developed to provide rapid and accurate diagnosis for patients presenting with acute undifferentiated chest pain [8]. Patients receive up to six hours of observation and biochemical testing followed by an exercise treadmill test. If these tests are positive then they are admitted to hospital with a clear diagnosis, if negative they are discharged home. Evaluation of CPU care has focussed upon cardiac events, process measures and economic measures [9]. There is some evidence that CPU care is associated with improved diagnostic certainty [10] and patient satisfaction [11], but no data to compare psychological morbidity and quality of life after CPU and routine care, despite substantial data to suggest that this is an important problem for patients [3-5,12-14].

The ESCAPE (effectiveness and safety of chest pain evaluation to prevent emergency admission) trial was a randomised controlled trial and economic evaluation of CPU versus routine care that showed that CPU care was associated with reduced hospital admission [15], improved health utility [15] and improved patient satisfaction [16], and was likely to be considered cost-effective [15]. This paper reports quality of life and psychological measures from the ESCAPE trial. We aimed to measure the effect of CPU care upon anxiety, depression, and health related quality of life, and to determine whether CPU care reduced subsequent symptoms of chest pain.

## Methods

The Northern General Hospital Emergency Department provides adult emergency care to the 530,000 population of Sheffield, United Kingdom. In 1999 a CPU was established in the emergency department, staffed by three specialist chest pain nurses, and able to accommodate up to six patients with acute undifferentiated pain. Patients were selected using validated clinical predictors and received two to six hours of observation and biochemical cardiac testing, followed by, where appropriate, an exercise treadmill test. Full details of the CPU protocol have been published [17]. Routine care, prior to development of the CPU, consisted of assessment by a doctor who had access to biochemical cardiac tests, but not observation facilities or exercise treadmill testing.

From 5^th ^February 2001 to 5^th ^May 2002 the CPU was subject to a cluster randomised controlled trial. Days of the week (N = 442) were randomised to CPU or routine care in equal numbers. All patients attending with acute chest pain were screened for eligibility in the trial. Patients were excluded if they had ECG changes diagnostic for an acute coronary syndrome, clinically obvious unstable angina, co-morbidity or alternative pathology requiring hospital admission (e.g. suspected pulmonary embolus), negligible risk of acute coronary syndrome (e.g. age less than 25 years), or if they were unable to consent to participation. Written, informed consent was requested and patients who agreed to participate were followed up in a review clinic at two days, and by postal questionnaire at one month. The study protocol was approved by the North Sheffield Research Ethics Committee. Full details of the ESCAPE trial have been published [15].

Health related quality of life was measured using the SF-36 questionnaire [18]. Anxiety and depression were measured using the Hospital Anxiety Depression Scale (HADS) [19]. Both are widely used, validated, self-completed questionnaires. Both were administered at two days and one month. At two days patients were handed the questionnaires in the review clinic and asked to complete it in their own time and return it to the Medical Care Research Unit. No reminder was sent to non-responders to this questionnaire. Further questionnaires were mailed at one month with one re-mailing for non-responders.

A brief additional questionnaire was sent at one month that was designed specifically for the study. This predominantly asked questions about health service use for the economic evaluation, but also asked participants whether they had suffered any further chest pain. If they responded that they had, they were asked to score the severity of the chest pain on a 100 mm visual analogue scale. A further question asked whether the patient had taken time off work since their hospital attendance.

The sample size estimate of 988 was based upon the primary outcome measure, the proportion of patients admitted to hospital. Assuming a response rate of 65% to the questionnaires, this sample size would provide 80% power to detect an effect size of 0.25 for these outcomes (alpha = 0.05). Using standard deviations derived from a two-week pilot study, this effect size equates to 1.1 points on the HADS anxiety or depression scores, 11.5 points of the SF-36 physical or emotional role dimensions, and 6 points on the other SF-36 dimensions. Data was analysed using Stata statistical software (version 8.0). Multi-level random effects modelling was used with day of week as a random effect to adjust for clustering by day of week. For the principal analysis no adjustment for confounding was made. For secondary analysis age, gender and past history of coronary heart disease were included as covariates (determined a priori to be important potential confounders), along with any variable that showed significant (p < 0.05) baseline imbalance between the study groups.

## Results

During the 442-day study period there were 6957 attendances with chest pain or a related complaint. Of these, 764 (11.0%) had ECG changes diagnostic for an acute coronary syndrome, 2402 (34.5%) had clinically obvious unstable angina, 869 (12.5%) had co-morbidity or alternative pathology requiring hospital admission, 1291 (18.6%) had negligible risk of acute coronary syndrome, and 513 patients (7.4%) were unable to participate in the trial or provide consent. The remaining 1118 patients (16.1%) were asked to participate in the trial and 972 agreed (86.9%). Response rates were: 717 (73.8%) to the initial questionnaire and 679 (69.9%) to the one-month questionnaire. The CONSORT diagram and full details of exclusions have been published elsewhere [15]. Baseline characteristics of the study groups are shown in Table 1. Source of referral, smoking status, and ECG at presentation showed significant baseline imbalance. Hence secondary analyses adjusted for these covariates, along with age, gender and past history of coronary heart disease.

**Table 1:** Baseline characteristics of the study groups

	**CPU care**	**Routine care**
Age (years)	49.4	49.6
Male sex (%)	304 (63.5%)	318 (64.5%)
Known CHD (%)	16 (3.3%)	27 (5.5%)
Hypertension (%)	127 (26.5%)	120 (24.3%)
Diabetes (%)	17 (3.5%)	29 (5.9%)
Hyperlipidaemia (%)	58 (12.1%)	70 (14.2%)
Smoker (%)	169 (35.3%)	143 (29.0%)
Family history (%)	189 (39.5%)	200 (40.6%)
**Pain nature**		
Indigestion / burning	60 (12.5%)	56 (11.4%)
Stabbing / sharp	116 (24.2%)	113 (22.9%)
Aching / dull / heavy	175 (36.5%)	181 (36.7%)
Gripping / crushing	66 (13.8%)	59 (12.0%)
Other	57 (11.9%)	71 (14.4%)
**Pain site**		
Central	317 (66.2%)	335 (68.0%)
Left chest	129 (26.9%)	125 (25.4%)
Right chest	19 (4.0%)	16 (3.2%)
Other	8 (1.7%)	8 (1.6%)
**Pain radiation**		
None	183 (38.2%)	189 (38.3%)
Left arm	118 (24.6%)	142 (28.8%)
Right arm	31 (6.5%)	26 (5.3%)
Neck	22 (4.6%)	22 (4.5%)
Jaw	15 (3.1%)	13 (2.6%)
Back	70 (14.6%)	53 (10.8%)
Other	27 (5.6%)	30 (6.1%)
**Pain duration**		
Continuous pain	312 (65.1%)	341 (69.2%)
Intermittent pain	93 (19.4%)	95 (19.3%)
**Other symptoms**		
Nausea	129 (26.9%)	161 (32.7%)
Vomiting	25 (5.2%)	31 (6.3%)
Dyspnoea	185 (38.6%)	202 (41.0%)
Sweating	192 (40.1%)	210 (42.6%)
**ECG at presentation**		
ECG normal (%)	412 (89.0%)	382 (82.2%)
ECG non-specific (%)	38 (8.2%)	64 (13.8%)
ECG old change (%)	13 (2.8%)	19 (4.1%)
**Source of referral**		
GP referral	138 (28.8%)	116 (23.5%)
Self referred	173 (36.1%)	155 (31.4%)
999	145 (30.3%)	189 (38.3%)
Other	23 (4.8%)	33 (6.7%)

Table 2 shows the final diagnosis recorded in the case notes, after hospital attendance and admission, of the most senior clinician to care for the patient. Those receiving routine care were more likely to have received a diagnosis of angina, whereas those receiving CPU care were more likely to have received a non-specific or non-cardiac diagnosis.

**Table 2:** Diagnostic impression after initial hospital attendance

**Diagnosis**	**CPU care**	**Routine care**
Non-specific chest pain	144 (30.1%)	125 (25.4%)
Anxiety	13 (2.7%)	21 (4.3%)
Angina	63 (13.2%)	123 (24.9%)
Myocardial infarction	28 (5.8%)	27 (5.5%)
Gastro-oesophageal pain	74 (15.4%)	60 (12.2%)
Musculo-skeletal pain	122 (25.5%)	106 (21.5%)
Other diagnosis	26 (5.4%)	18 (3.7%)
Not recorded	9 (1.9%)	13 (2.6%)

P < 0.0001 for the difference in distribution across the categories

Table 3 shows the mean SF-36 scores for both groups at two days, with the adjusted difference, 95% confidence interval, p-value and intraclass correlation coefficient. Table 4 shows these estimates at one month. At two days, CPU care was associated with significant improvements in physical functioning, vitality and general health. At one month, CPU care was associated with significant improvements in all dimensions of quality of life, except the emotional role dimension.

**Table 3:** Mean SF-36 scores at two days

	**N (% completed)**	**CPU care**	**Routine care**	**Difference**	**95% CI**	**P-value**	***ρ***
							
				Unadjusted			
							
				Adjusted			
Physical functioning	694 (96.7%)	74.8	69.7	5.1	1.1 to 9.0	0.012	0.002
				4.2	0.4 to 7.9	0.029	
Social functioning	703 (98.0%)	72.2	69.8	2.4	-1.7 to 6.6	0.252	0
				1.5	-2.7 to 5.6	0.49	
Role-physical	684 (95.4%)	50.4	46.0	4.4	-2.2 to 11.0	0.191	0.028
				3.3	-3.3 to 10.0	0.326	
Role-emotional	685 (95.5%)	64.7	59.5	5.2	-1.2 to 11.6	0.113	0
				5.1	-1.2 to 11.4	0.111	
Mental health	700 (97.6%)	66.9	64.7	2.2	-0.9 to 5.3	0.158	0
				2.3	-0.7 to 5.4	0.132	
Vitality	697 (97.2%)	52.3	47.6	4.6	1.3 to 8.0	0.007	0
				4.6	1.3 to 8.0	0.007	
Pain index	701 (97.7%)	50.8	49.0	1.8	-1.9 to 5.5	0.351	0
				2.0	-1.7 to 5.7	0.284	
General health	688 (96.0%)	60.3	54.5	5.7	2.3 to 9.2	0.001	0
				5.4	2.0 to 8.8	0.002	

Upper row shows unadjusted analysis (primary analysis) Lower row shows adjusted analysis (secondary analysis) *ρ *= Intraclass correlation coefficient. This provides a measure of the amount of clustering of each outcome by the unit of randomisation (day).

**Table 4:** Mean SF-36 scores at one month

	**N (% completed)**	**CPU care**	**Routine care**	**Difference**	**95% CI**	**P-value**	***ρ***
							
				Unadjusted			
							
				Adjusted			
Physical functioning	654 (96.3%)	74.1	66.2	7.8	3.8 to 11.9	<0.001	0.025
				7.6	3.6 to 11.5	<0.001	
Social functioning	654 (96.3%)	74.6	67.0	7.6	3.2 to 12.0	0.001	0
				6.8	2.4 to 11.2	0.002	
Role-physical	638 (94.0%)	54.1	46.0	8.2	1.3 to 15.0	0.02	0
				7.0	0.4 to 13.6	0.039	
Role-emotional	630 (92.8%)	63.9	60.2	3.7	-3.0 to 10.5	0.281	0
				3.9	-2.8 to 10.5	0.256	
Mental health	653 (96.2%)	69.1	64.4	4.7	1.3 to 8.2	0.007	0
				5.2	1.9 to 8.6	0.002	
Vitality	649 (95.6%)	52.6	47.1	5.5	1.8 to 9.2	0.003	0
				5.8	2.2 to 9.3	0.002	
Pain index	655 (96.5%)	66.4	62.0	4.4	0.2 to 8.5	0.04	0
				4.3	0.2 to 8.3	0.041	
General health	651 (95.9%)	59.7	51.7	8.0	4.6 to 11.5	<0.001	0
				8.1	4.6 to 11.5	<0.001	

Upper row shows unadjusted analysis (primary analysis) Lower row shows adjusted analysis (secondary analysis) *ρ *= Intraclass correlation coefficient

**Figure 1:**
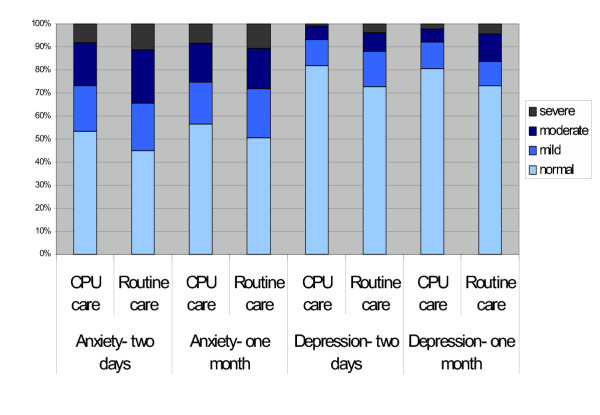
HADS scores categorised according to severity

Table 5 shows the summary HADS data at two days and one month. CPU care was associated with lower depression scores at both two days and one month. An early significant reduction in anxiety associated with CPU care was no longer significant at one month. HADS data is also summarised in the Figure 1, categorised according to severity of anxiety and depression. Scores of zero to seven are normal, eight to ten are mild, eleven to fourteen are moderate, and fifteen to twenty-one are severe. Most participants had normal levels of depression, but only half reported normal levels of anxiety. CPU care was associated with increased prevalence of normal levels of anxiety at two days (53.4% vs 45.1%; p = 0.028) but not at one month (56.7% vs 50.8%; p = 0.129), and increased prevalence of normal levels of depression at two days (81.8% vs 72.9%; p = 0.005) and one month (80.4% vs 73.2%; p = 0.029).

**Table 5:** Mean HADS scores at two days and one month

	**N (% completed)**	**CPU care**	**Routine care**	**Difference**	**95% CI**	**P-value**	***ρ***
							
				Unadjusted			
							
				Adjusted			
Anxiety- two days	702 (97.9%)	7.73	8.62	0.89	0.21 to 1.56	0.01	0
				0.75	0.09 to 1.41	0.027	
Depression-two days	701 (97.8%)	4.30	5.23	0.93	0.34 to 1.51	0.002	0
				0.84	0.26 to 1.42	0.005	
Anxiety-one month	645 (95.0%)	7.29	7.77	0.48	-0.26 to 1.23	0.203	0
				0.58	-0.15 to 1.31	0.117	
Depression-one month	644 (94.8%)	4.42	5.43	1.00	0.36 to 1.66	0.002	0
				1.02	0.37 to 1.66	0.002	

Upper row shows unadjusted analysis (primary analysis) Lower row shows adjusted analysis (secondary analysis) *ρ *= Intraclass correlation coefficient

At one-month follow-up, 143 out of 318 participants (45.0%) receiving CPU care reported having further pain, compared to 168 out of 314 (53.5%) receiving routine care (unadjusted OR for further pain if receiving CPU care = 0.71, 95% CI 0.52 to 0.97, p = 0.032; adjusted OR = 0.65, 95% CI 0.10 to 0.76, p = 0.010). For those reporting further pain, the mean score on a 100 mm visual analogue pain score was 36.5 mm among those receiving CPU care and 43.0 mm among those receiving routine care (unadjusted difference = 6.5 mm, 95% CI 2.2 to 10.8, p = 0.003; adjusted difference= 6.8 mm, 95% CI 2.2 to 11.5, p = 0.004). Thus, at one month, CPU care was associated with a reduction in the incidence and severity of subsequent chest pain.

One month after hospital attendance, 49 out of 315 participants receiving CPU care (15.6%) reported that they had taken time off work, compared to 58 out of 316 receiving routine care (18.4%). The unadjusted odds ratio for taking time off work after receiving CPU care was 0.82 (95% CI 0.54 to 1.24, p = 0.35; adjusted OR 0.79 (95% CI 0.59 to 1.22, p = 0.287).

## Discussion

### Main findings

Patients with acute, undifferentiated chest pain who received CPU care had improved quality of life and reduced psychological symptoms. All dimensions of quality of life were improved at one month apart from the emotional role dimension. Anxiety was reduced two days after assessment, but there was no significant difference by one month, whereas reduced symptoms of depression at one month were still significant at one month. Patients receiving CPU care reported that subsequent symptoms of chest pain were less frequent and (if present) less severe. However, these reported differences in symptoms and quality of life were not associated with any significant difference in the need to take time off work.

### Comparison to other studies

Previous studies of CPU care have focussed on cardiac events, process measures and economic measures [9]. One previous randomised trial found that CPU care was associated with greater diagnostic certainty [10] and improved patient satisfaction [11]. Our study suggests a more complicated picture, since more patients in the CPU group received a diagnosis of non-specific chest pain. CPU assessment may allow cardiac disease to be ruled out, but if an alternative diagnosis is not offered then this can hardly be said to increase diagnostic certainty, except in the somewhat convoluted sense that we may be more certain of what we know the cause is not.

Nevertheless, CPU assessment was associated with reduced anxiety and improved quality of life. This is consistent with a previous study of diagnostic testing by Sox et al [20] that showed reduced anxiety among patients who were randomised to a more thorough outpatient diagnostic work-up for non-specific chest pain, but inconsistent with the findings of a study of exercise testing by Channer et al [21] that found no evidence of reassurance.

### Limitations of this study

The main limitation of this study relates to our inability to blind participants to the intervention they received and to fact that they were involved in a trial of CPU care. Participants may have been influenced by this knowledge and improvements in psychological symptoms and quality of life may represent a positive response to receiving a novel form of care, rather than improvements specifically related to CPU care.

The use of cluster randomisation has substantial advantages for pragmatic evaluation of changes in organisation, particularly if economic evaluation is undertaken [22]. However, the fact that randomisation occurs before recruitment means that there is the potential for selection bias. We attempted to reduce this risk by applying rigorous selection criteria and to address any potential bias by undertaking a secondary, adjusted analysis. Nevertheless it is possible that selection bias may have influenced the results.

Although the measures used have been validated, they have not been widely used in the emergency setting. Changes in health status after an episode of chest pain may be very rapid, hence our need to measure outcomes only two days after intervention. Yet the HADS measures anxiety and depression over the previous week, while some SF-36 questions refer to the previous month. A recent episode of chest pain is likely to be an important determinant of reported health, but it may be that, if participants interpreted the questionnaires strictly, the initial questionnaire was recording health status before the intervention. Also, there may be doubts regarding what some of the outcomes are actually measuring. For example, some of the questions in the HADS measure symptoms that are useful markers for depression, such as levels of activity, which may also be changed by other health or social processes. Thus it may be that the reduced scores associated with CPU care measured on the depression scale relate to increased activity in response to the CPU exercise treadmill test, rather than reduced depression.

### Implications for practice and future research

This study suggests that the assessment that patients receive when they present with acute chest pain can have an impact upon their subsequent health, even if this assessment does not, in most cases, provide a definitive diagnosis. It supports the findings of decision analysis modelling [7] that the potential health impact of chest pain assessment lies as much in addressing quality of life and psychological symptoms as in detecting and treating cardiac disease. The CPU assessment simply provides a rigorous and structured evaluation, yet this appears to have a significant effect upon anxiety (although this is not maintained), depression and quality of life.

Yet it is not clear how this effect is achieved. It is possible that early, rigorous testing, particularly the exercise treadmill test, has a valuable effect in reassuring the patient that they are healthy and capable of normal physical functioning. Alternatively, it could be that consistent, reliable advice and attention from specialist chest pain nurses, rather than a variety of different doctors, is the key element. A third possibility, as previously discussed, is that bias plays an important role.

Future research needs to determine which of these possibilities is the key factor. This is important for the specific issue of determining whether and how CPU care is effective, and thus what elements of CPU care are essential, and for the more general issue of exploring how diagnostic assessment effects subsequent well being.

## Conclusions

CPU care for patients attending hospital with acute, undifferentiated chest pain is associated with reduced initial anxiety, reduced depression over the following month, and improvements in most dimensions of quality of life. Further research is required to establish how this effect is achieved.

## List of abbreviations

OR: odds ratio

CI: confidence interval

ECG: electrocardiograph

CPU: chest pain unit

ESCAPE: effectiveness and safety of chest pain assessment to prevent emergency admission

HADS: hospital anxiety depression scale

## Authors' contributions

SG conceived and designed the study, analysed the data, drafted the paper, and participated in writing the final paper. JN assisted with study conception and design, supervised data analysis, and participated in writing the final paper.
